# Smart and Portable Air-Quality Monitoring IoT Low-Cost Devices in Ibarra City, Ecuador

**DOI:** 10.3390/s22187015

**Published:** 2022-09-16

**Authors:** Vanessa E. Alvear-Puertas, Yadira A. Burbano-Prado, Paul D. Rosero-Montalvo, Pınar Tözün, Fabricio Marcillo, Wilmar Hernandez

**Affiliations:** 1 Computer Science Department, University of Salamanca, 37008 Salamanca, Spain; 2 Electronic Department, Instituto Superior Tecnológico 17 de Julio, Ibarra 100102, Ecuador; 3 Computer Science Department, IT University of Copenhagen, 2300 Copenhagen, Denmark; 4 Department of Computer Architecture and Technology/CITIC, University of Granada, 18071 Granada, Spain; 5 Facultad de Ingenieria y Ciencias Aplicadas, Universidad de Las Americas, Quito 170513, Ecuador

**Keywords:** internet of things, environmental monitoring, air-quality measurement, machine learning application, data analysis

## Abstract

Nowadays, increasing air-pollution levels are a public health concern that affects all living beings, with the most polluting gases being present in urban environments. For this reason, this research presents portable Internet of Things (IoT) environmental monitoring devices that can be installed in vehicles and that send message queuing telemetry transport (MQTT) messages to a server, with a time series database allocated in edge computing. The visualization stage is performed in cloud computing to determine the city air-pollution concentration using three different labels: low, normal, and high. To determine the environmental conditions in Ibarra, Ecuador, a data analysis scheme is used with outlier detection and supervised classification stages. In terms of relevant results, the performance percentage of the IoT nodes used to infer air quality was greater than 90%. In addition, the memory consumption was 14 Kbytes in a flash and 3 Kbytes in a RAM, reducing the power consumption and bandwidth needed in traditional air-pollution measuring stations.

## 1. Introduction

Environmental pollution is an issue that has undeniably attracted our full attention. The problem of air pollution affects people’s physical and mental health, and long-term exposure increases the risk of cardiovascular and respiratory diseases [1]. In addition, the World Health Organization (WHO) forecasts that 4.2 million people die every year because of exposure to air pollutants. This concern is more evident in urban sectors with high population density, since their air-pollution levels have increased [2]. Indeed, cities represent only about 2% of the geographic area and accommodate over 50% of the world’s population [3]. Therefore, it is necessary to describe human behavior to detect where and when traffic increases and people are at greater risk of air-pollution exposure [4]. Thus, governments can receive relevant information to propose new transport policies/alternatives that are adjusted to the specific characteristics of each city [5].

Following this environmental concern, several initiatives from worldwide organizations have proposed limiting the emission of harmful gases that come from the combustion of fuels, especially petroleum [6]. For this reason, one of the most relevant proposals in this field is the Paris Agreement, because it had several countries commit to reducing the production of polluting gases [7]. Its main objective in environmental terms is to limit the increase in global temperature to below two degrees Celsius per year. In fact, if the global temperature exceeds this value, this could have an irreversible impact on the environment and could affect all ecosystems on the planet [8].

Therefore, from a traditional point of view, environmental protection agencies have started to set up fixed-site air-quality monitoring stations in many regions to collect data on air-quality conditions. Additionally, nonprofit organizations, such as waqi.org (https://waqi.info/ accessed on 17 August 2022), provide information on current air pollution using more than 30,000 monitoring stations installed in 2000 cities around the world. However, in Latin America [9], several cities do not have air-quality monitoring stations. For example, in Ecuador, there is currently only one air-quality measurement node located in Cuenca, and nine in Quito [10]. For this reason, the commitment made by some countries to limit their emissions of polluting gases has become a challenging task. This is because of the following two reasons: (a) traditional air-quality stations do not have the necessary infrastructure to acquire data on environmental pollution [11], and (b) these stations are rigid and, consequently, fixed installation points can only represent approximations of the phenomenon [12]. For these reasons, low-cost sensors are a suitable solution to deploy data acquisition systems and, combined with conventional equipment, allow air quality to be monitored more effectively [13]. In addition, low-cost sensors are part of an embedded system (i.e., sensors, microcontroller, and battery) and are capable of sending data by means of different communication protocols. In this way, they become Internet of Things (IoT) devices [14].

The main characteristics of low-cost sensors can be summarized as follows: ease of deployment, fast integration of several sensors with low power consumption, and their flexibility to be installed in remote locations [9]. However, due to their interaction with the environment, IoT low-cost devices can suffer from malfunctions caused by environmental conditions or deterioration of their materials [15]. On the other hand, due to the exponential use of IoT devices, their development in recent years has improved their data processing capability, power consumption, and various long-range wireless technologies for sending data. Consequently, today, these devices have enough computational resources to implement machine learning (ML) model inference aimed at local decision making [16]. Therefore, some trends, such as federated learning, allow complex algorithms to be compiled based on their input data on end devices such as tablets, phones, and, specifically in this case, electronic devices [17]. It also ensures that data are processed locally and avoids the risk of being intercepted. In addition, it reduces the processing load on servers due to massive data sending [8]. Nevertheless, it is necessary to determine the random-access memory (RAM) needed to compile a robust application that enables secure processing and avoids remote attestation [18].

Taking into account everything stated above, this research proposes the development of low-cost smart, portable IoT devices for air-quality monitoring. These devices will be installed in public and private vehicles in Ibarra, Ecuador, to collect the required information to describe the air-pollution phenomenon. To do so, first, we design an electronic system that collects data while having the ability to detect outliers [5]. Then, with the data sent to an external server, we will train several supervised learning models to determine which one best describes the studied phenomenon. Later, the algorithm with the highest classification performance and lowest computational cost will run on the IoT device to infer the class of new incoming data.

The implementation of classification algorithms helps provide relevant information for decision making. In this paper, through labels, a heat map of the city is represented in accordance with the pollution indexes and the areas of high vehicular traffic. This is carried out together with the concentration of gases. With this information, we can validate whether the policies of government entities meet the objective of reducing emissions of polluting gases. In addition, citizens can choose to take alternative routes so as not to be exposed to areas with a high concentration of air pollutants. When mentioning that the system infers the class of the new data, it means that the nodes have the ability to make decisions locally, freeing up computational cost on the server and avoiding latencies. Additionally, once the classification is implemented in its memory, the system can determine and classify the pollution indexes at any time of day. Established classes are shown in upcoming sections of the paper.

In short, the classification benefits citizens, because they can now access the required information and observe the heat map of the city, with respect to the concentration of polluting gases. Likewise, the classification allows researchers in the field of polluting gases analysis to compare the results obtained with a system that detects local patterns—that is, in the place where the measurement is carried out. In other words, the device’s decision means it is not necessary to constantly perform analyses from the server, which consumes much more energy and computational power.

Finally, a user interface (GUI) is available on a cloud server in order to store relevant data to improve the model and display the environmental pollution of the city in a heat map. As a result, One-Class Support Vector Machine (One-Class SVM) is defined as an anomaly detection algorithm used to eliminate outliers. In terms of classification algorithms, we had similar results with the Decision Tree algorithm and Neural Networks, with consumption of 12 Kbytes of flash and 3 Kbytes in RAM, with a processing time of approximately 1 s.

In short, the novelty of this research is the presentation of an IoT architecture used to deploy ML models locally, using low-cost sensors to reduce the power consumption and bandwidth needed to process large datasets in the Cloud. Therefore, the main contributions of this paper are as follows:

- We present an extensive literature review to select the suitable low-cost sensors available to collect air-pollution data properly.
- We design an IoT architecture showing characteristics of the transmission channel, the type of database used, and the corresponding data analysis tasks needed to run ML models close to the end-user.
- Robust data analysis based on ML techniques is presented with stages of data acquisition and data preprocessing, such as: (a) outlier detection, (b) classification model building, and (c) tests that are necessary to work in natural environments. Here, this analysis has been applied to contribute to the solution of current concerns such as environmental pollution.
- A computational cost analysis is performed to define suitable ML algorithms for IoT devices and the new challenges of implementing them in devices with limited processing capabilities.

This paper has been organized as follows. Section 2 presents a background on air-quality indexes and related works. Section 3 shows the design of the IoT device. The proposed architecture is shown in Section 4. The data analysis is conducted in Section 5. Section 6 presents the results. Finally, the conclusions are given in Section 7.

## 2. Background

This section shows relevant information on air-quality indexes of gases that affect the health of people, and related works.

### 2.1. Air-Quality Indexes

The Air-Quality Index (AQI) is a quantitative measure that defines the air-quality condition in a specific site. Therefore, there are established levels defined by the risk of gas concentration and how they affect human health [17]. AQI consists of measurements from 0 to 500, with the highest value of more significant concern for the level of air pollution and greater health risk. Traditionally, countries obtain air-pollution data daily and create a relationship between the maximum and minimum concentration to establish the AQI between good, moderate, unhealthy for sensitive groups, unhealthy, very unhealthy, and hazardous. The gases taken into account are Sulfur Dioxide (SO${}_{2}$), Nitrogen Dioxide (NO${}_{x}$), Ozone (O${}_{3}$), and Carbon Monoxide (CO) [1,10,19,20,21]. However, as has been witnessed in several papers [14,22,23], there is no metric of ambient air pollution that includes related factors such as temperature, relative humidity, and ultraviolet (UV) exposure. In addition, having daily samples of the above-mentioned variables allows government entities to find similarities within human activities. For this reason, we have defined a pollution traffic light (red, yellow, and green) that includes the previously cited variables that specifically describe the environmental conditions of the city of Ibarra, Ecuador. Consequently, we start by generalizing a model for the AQI levels to a specific solution for Ibarra. This is carried out based on the population and vehicle density of this city.

### 2.2. Related Works

In recent years, electronic devices have increasingly developed the number of air-quality monitoring systems placed in cities. Novel IoT solutions, such as the one shown in [4,24,25], presented works where electronic systems send data to cloud computing to measure the air-quality index. In [14,26], the authors implemented classification algorithms such as the Decision Tree algorithm and data clustering on external servers, once wireless sensor networks acquired the dataset. Subsequently, ref. [5] focuses on sensor calibration techniques in different wireless sensor network nodes for a correct data acquisition process. In addition, in [2,9,22] the authors focused on the design of electronic systems that are part of IoT architectures, and can send data using edge computing platforms for data preprocessing (cleaning) stages. Furthermore, these electronic systems have interfaces to cloud servers for constant data monitoring and sending messages in the event of unexpected events.

In a work related to wireless protocols and sending data over long distances, Firdaus et al. [27] presented an indoor air-quality monitoring system using IoT and LPWAN LoRa as communication systems. This proposal uses temperature, humidity, CO and CO${}_{2}$ sensors. Then, the information is sent to a server in the cloud using the LPWAN LoRa communication protocol to be visualized in an Android application. Additionally, Ali et al. [12], show a proposal for low-cost sensors based on LoRaWAN networks for air-quality measurement. This work proposes implementing three nodes, each with five sensors and the ability to connect over the long-range network using the LoRaWAN communication protocol. The sensing parameters are as follows: NO${}_{x}$ (i.e., NO + NO${}_{2}$), CO, particulate matter, temperature, humidity, and gases. The system also consists of solar-charged lipo batteries. As a result, air-pollution forecasting models were developed during data acquisition using ML techniques.

Recently, works such as [23,28,29] focused on reducing the amount of data by optimizing and calibrating low-cost sensors correctly, to collect rich data used to train ML models. Lastly, Andrade et al. [30] present a novel alternative to developing tiny ML models to forecast values of CO${}_{2}$ emissions over time.

The above-mentioned works show that there is a trend to improve IoT low-cost devices by means of data preprocessing techniques and light ML models. However, there are still open problems in developing an IoT architecture, where IoT devices can make decisions locally and reduce both bandwidth and power consumption. Moreover, determining the RAM/Flash needed to create ML models is a relevant aspect to take into consideration, because it provides the new functionalities that emerging microcontrollers must have.

## 3. IoT Device Design

This section presents the design of the IoT device, starting with the selection of sensors. Then, the calibration of sensors is shown and, finally, the voltage supply and rain protection are described.

To select the correct air-pollution gases, Alit et al. [12] presented an electronic system by selecting several sensors from many brands and different signal conditioning stages. They demonstrated the necessity of comparing works to define the suitable sensors that are needed to deploy an IoT device. On the other hand, Refs. [11,15] presented solutions by implementing ML algorithms using external databases that were obtained in the United States and Europe. As a result, they presented a comparison of several ML classification algorithms that fit an air-pollution analysis. Therefore, we used this information to design the IoT device by comparing sensors and ML algorithms.

### 3.1. Sensor Selection

Table 1 presents some relevant works, their technology, and the summary of the sensors they used. These works provide a comparative evaluation of sensors, microcontrollers, and communication protocols. Consequently, the sensors for CO, NO${}_{x}$ and CO${}_{2}$ gases derived from the poor combustion of fossil fuels are established (respectively, NO${}_{x}$ for diesel, and CO${}_{2}$ gases for gasoline [19]). In addition, temperature and humidity data are used to describe human behavior, which is very relevant to this study. Furthermore, to achieve higher coverage and mobility of IoT devices, and considering that in Ecuador, there is no backbone of the LoRa network that IoT devices can connect to to send data, a GPS/GPRS communication is defined due to its extended coverage.

Several brand new sensors are available to deploy air-quality stations. Precision and accuracy are relevant in selecting a suitable sensor, especially for gas concentration. However, there are other requirements to consider, such as size and communication protocols. Therefore, sensors such as Envio+ (https://www.switch-science.com/catalog/6119/ accessed on 17 August 2022), SDS011 (https://aqicn.org/sensor/sds011/ accessed on 17 August 2022), and OPC-R1 (https://www.isweek.com/product/pm2-5-particle-sensor-opc-r1_2315.html accessed on 17 August 2022), related to the IoT device proposed are not a good alternative, even when some functionalities are superior to the MQ series and Alphasense sensors, which are selected to deploy them into the IoT device. Unfortunately, Alphasense sensors were unavailable at the time to acquire the hardware needed. Furthermore, Kurenshi et al. [31], mention that any low-cost sensor could improve its robustness via regression and ML algorithms using a reference-grade or research-grade instrument. Consequently, even when MQ sensors do not have the highest precision, their performance might be improved significantly, as demonstrated in the following sections [32].

Therefore, the selected sensors are as follows: MQ-135 for NO${}_{x}$ analysis, MQ-7 for CO, SCD30 for temperature, humidity and CO${}_{2}$ data collection, and VLM6075 for UV detection. Moreover, the SIM 808 module is used to send data by GPS/GPRS protocol. These sensors were selected based on functionality, features, and usability requirements. An Arduino nano BLE sense is used as the electronic board. This board uses an nRF52840 Harvard architecture microcontroller from the ARM cortex M4 family. The IoT device is programmed in the Arduino environment (i.e., C language).

### 3.2. Calibration of Sensors

As part of the sensor calibration process, it is necessary to analyze the different sensitivity curves provided in the corresponding data sheets. In addition, within the process, a review of the state of the art was carried out to determine the most effective methods for an adequate calibration and reading of the sensors. In this work, we have proceeded as follows.

Due to the fact that MQ sensors have been built with sensitive materials used to detect different concentrations and types of gases, their data sheets specify the calibration process by using the load ($R_{L}$) and target gas ($R_{S}$) resistors, and the ratio of the sensor resistance in clean air over the resistance of the sensor in various gases ($R_{O}$). Therefore, to obtain $R_{S}$, it is necessary to use the voltage supply ($V_{C}$) and the voltage that the sensor receives ($V_{RL}$). The equation used is (1).

(1) $$R_{S}=\frac{V_{c}-V_{RL}}{V_{RL}}\cdot R_{L}$$

Each MQ sensor has a sensitivity curve where the *x*-axis is the detected concentration of the gas in parts per million (ppm), while the *y*-axis is the $R_{S}/R_{O}$ ratio. The MQ7 sensor was selected to measure the concentration of CO. According to the manufacturer’s data sheet, this sensor can detect concentrations from 20 ppm to 2000 ppm. From the sensitivity curve, the following parameters are considered for the sensor reading: (1) temperature: 20 ${}^{{}^{\circ}}$C; (2) humidity: 65%; (3) O${}_{2}$ concentration: 21%; and (4) a value of $R_{L}=10$ kΩ. $R_{0}$ is the resistance value at 100 ppm of CO in clean air, and $R_{S}$ is the resistance to different gas concentrations. To obtain the ppm value, we worked with (2).

(2) $$\mathrm{CO}(\mathrm{ppm})=10\cdot \frac{log\left(17.5\right)-log\left(\frac{R_{S}}{R_{0}}\right)}{0.63}$$

To detect NO${}_{x}$, the chosen sensor was MQ135, with parameters as follows: temperature: 20 ${}^{{}^{\circ}}$C; humidity: 65%; O${}_{2}$ concentration: 21%; and a value of $R_{L}=20$ kΩ, where $R_{0}$ is the resistance value at 100 ppm of NO${}_{x}$ in clean air, and R${}_{S}$ is the resistance to different gas concentrations. To obtain the ppm value, we worked with (3).

(3) $${\mathrm{NO}}_{x}\left(\mathrm{ppm}\right)=156-133\cdot \frac{R_{S}}{R_{0}}$$

The VLM6075 UV sensor has a photodiode that measures ultraviolet (UV) radiation levels, A (320–400 nm) and B (280–320 nm), allowing the calculation of the UV index with a variation of ±10 nm, and that sends information using I${}_{2}$C communication, with a resolution of 16 bits. On the other hand, for the measurement of CO${}_{2}$, the accuracy of the SCD30 sensor is ±30 ppm ±3% (25 ${}^{{}^{\circ}}$C, 400–10,000 ppm). The humidity has a variability of 3% on a scale from 0 to 95%, and the temperature has a variability of 0.5 ${}^{{}^{\circ}}$C, with measurements up to 70 ${}^{{}^{\circ}}$C. Those sensor are digital and have auto-calibration techniques in their libraries.

**Figure 1:**
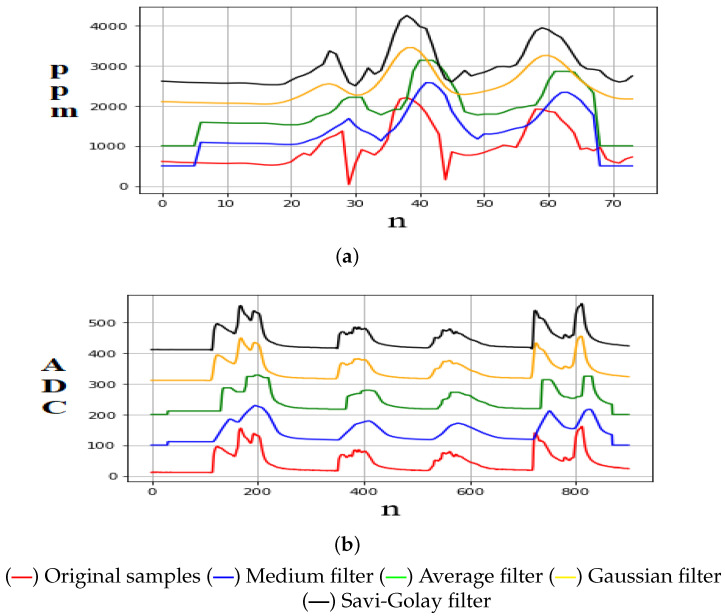
Signal smoothing analysis. (**a**) CO${}_{2}$ measurement by using the SCD30 sensor. The *y*-axis is the sensor output in ppm, and the *x*-axis is the number of samples. (**b**) NO${}_{x}$ measurement by using the MQ-135 sensor. The *y*-axis is the sensor output given by a 10-bit analog-to-digital (ADC) converter (1024 discrete levels). The *x*-axis is the number of samples.

Finally, samples were taken by exposing each sensor to its magnitude to observe its errors and define the correct sampling time. Kowalski et al. [33] mentioned that the most relevant signal smoothing filters (with variable frequency) are as follows: Average, Median, Gaussian, and Savitsky–Golay filters. For this reason, in this research, samples were taken from each sensor to apply these filters. In addition, using the signal-to-noise ratio (SNR) metric, here it is shown which of them eliminates the erroneous components inserted in the signal [34]. Furthermore, it was observed that the average filter is adequate to be implemented by taking *n* samples with a window of size *k* = 25. Figure 1 shows the signal smoothing obtained by applying the above filters.

### 3.3. Voltage Supply and Rain Protection

The system is placed on the roof of the vehicle and is magnetically fastened. When the key of the vehicle is turned, this action closes the starting circuit, the motor engine starts, and the alternator comes on to supply power to the vehicle and power the system with 12 V, which requires rectification to 5 V. Next, the IoT device turns on and leaves its rain protection case to begin data collection. If the system detects rain or the vehicle turns off, it returns to its initial position inside its case.

## 4. IoT Architecture

IoT devices have limited computational resources. Therefore, it is necessary to work with lightweight network protocols and services, which are also oriented to the type of information being sent [18]. For this reason, the message sending protocol is the Message Queuing Telemetry Transport (MQTT), because it has a variable light payload messaging service with a publisher/subscriber model [22]. Consequently, Mosquitto (https://mosquitto.org/ accessed on 17 August 2022) is used as the server. Furthermore, following the lightweight computational footprint, the database should not be relational because the information is not concatenated with other information sources. Thus, a time-series database called InfluxDB (https://www.influxdata.com/ accessed on 17 August 2022) is used. The data will be stored for each node and topic (named for each variable in MQTT). In summary, the functionality of each block of the proposed IoT architecture is described as follows:

- **IoT node:** Collect sensor data and send messages via MQTT to the edge server.
- **Edge:** Has the MQTT broker, which receives data from IoT nodes. Then, it stores data in a time series database identifying each node.
- **Cloud:** We use a public data viewer called Grafana (monitoring stack) to securely connect to the database allocated in the edge and make querying from the cloud side.

**Figure 2:**
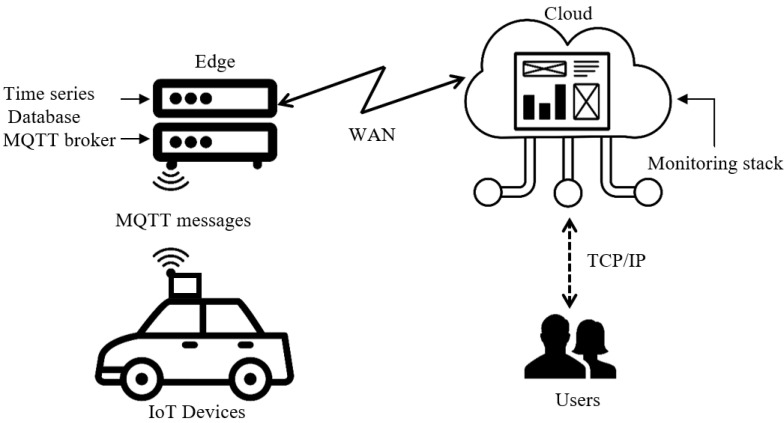
Proposed IoT architecture.

Figure 2 shows the IoT architecture. Users are considered vehicles and people who can observe and obtain reports.

## 5. Data Analysis

This section presents the data analysis scheme used to locally implement the ML algorithm. It is essential to consider that, in Ibarra, it is known from previous studies that the air quality is acceptable [1,4,10]. However, it shows punctual peaks that generate risk for people.

### 5.1. Original Data

The data are collected once the IoT device is built with the appropriate sensor calibration. In addition, high-level systems are used to ensure that the data are reliable, such as Air Quality Station in conjunction with MaxiMet weather stations GMX-240 from Libelium (https://www.libelium.com/iot-solutions/smart-cities/ accessed on 17 August 2022), and mobile applications that receive information from the different satellites that surround the globe. To describe the air-pollution phenomenon, seven specific data collection schedules (information obtained from government agencies) were established according to vehicle density and the hours of highest traffic flow (8h00, 13h00, and 17h00), normal traffic flow (10h00, 13h00 and 20h00), and reduced traffic flow (2h00). The data acquisition process starts taking 50 samples every two minutes in the above-mentioned schedules, while the vehicle is driven around the city for approximately two months. Additionally, due to the warming-up condition of low-cost sensors, the system waits 5 min to start taking samples. Furthermore, the smoothing algorithms and outlier detection techniques prune incorrect data, improving the quality of the dataset. After that, the data are sent to the InfluxDB database. In this case, the number of samples sent to InfluxDB is equal to 140,000. Moreover, each obtained datum has been classified according to the defined schedule.

At this point, it is important to mention that in this paper, the data set was divided according to the similarity of the values of the different variables into subsets. Therefore, we call classes to these subsets of data, as is carried out in machine learning. For us, a class is a set of data that have the same characteristics. Thus, once the classes were defined, algorithms were trained to generate rules that associate new data with the corresponding class. That said, in the event that there are values close to two classes, the algorithm makes its decision based on the variable that has the greatest weight or significance. In this way, the data are classified by criteria or functions defined by the algorithm itself.

Taking into account everything said above, the measurements of each variable are now categorized within different air-pollution levels (i.e., classes), which are established by government air-quality measurement networks (e.g., see Quito Metropolitan Network of Atmospheric Monitoring reports (QMNAM) at http://www.quitoambiente.gob.ec/index.php/informes accessed on 17 August 2022). In this paper, following QMNAM reports, the abovementioned classes were defined as follows:

- **Class A**: High levels of pollution with increased incidence of UV rays and high temperatures.
- **Class B**: Acceptable levels of pollution and moderate temperature.
- **Class C**: Low levels of gas concentration and suitable environmental conditions.

It is important to mention that the National Transit Agency of Ecuador imposes the maximum speed in cities at 50 km/h. In addition, the IoT application is focused on collecting data in city zones with high vehicle density, which reduces the chance that the IoT device has inaccurate measures by medium/high vehicle speeds. Furthermore, smoothing algorithms eliminate those errors by comparing samples taken with the same sample rate.

### 5.2. Outlier Detection

**Figure 3:**
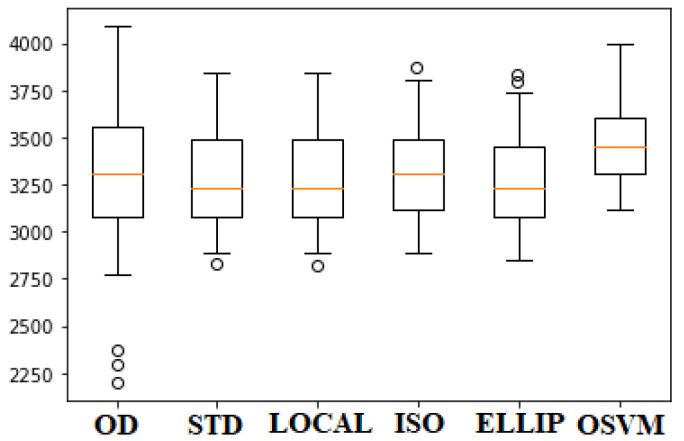
Box plot of data from the outlier detection methods.

Due to the nonlinearity of the electronic elements and sensor wear, errors appear in the data that may not follow the same distribution and do not have the same trend as the important information. For this reason, they can impair the data acquisition process [35]. Therefore, descriptive unsupervised learning techniques allow elimination of these data in order to find a refined training set. Consequently, the most relevant techniques found in the literature review are as follows: Standard Deviation, Local Outlier Factor, Isolation Forest, Elliptic Envelope, and One-Class SVM [36]. However, it is necessary to know which of the above-mentioned methods suitably fits the data type of the proposed system. Therefore, knowing the statistical distribution of the original samples allows detection of outliers based on quartiles. Figure 3 shows the box plot of the original data set (OD) of the CO variable, and how unsupervised learning techniques prune data that have different distributions. As a result, it can be observed that the anomaly detection algorithms allow the data to be concentrated towards a central tendency while eliminating the distant ones (Gaussian bell). Therefore, it is observed that the Standard Deviation (STD) and Local Outlier Factor (LOCAL) algorithms have similar results. Furthermore, Isolation Forest (ISO) and Elliptic Envelope (ELLIP) algorithms maintain outliers in their corresponding dataset. Finally, One-Class SVM (OSVM) shows the lowest data variability and has no outliers.

### 5.3. Supervised Classification

There are several methods of supervised learning that use different mathematical approaches. These approaches define the model complexity and determine the memory footprint. For this reason, it is necessary to carry out a performance test to determine a suitable method. Consequently, the methods used are as follows: based on distances (k-NN), probabilities (Naive Bayes), following the models (SVM), heuristic (Decision Tree), and deep learning (Neural Networks).

Here, the cross-validation metric is used to determine the classification performance, randomly dividing the original data set into two subsets. The first is the training subset, which allows the creation of a classification model. The second subset allows us to check if the prediction made by the model is correct by verifying whether the label prediction matches the one assigned to the data acquisition process. Thus, by performing this process several times, a balanced performance is achieved in the model, because it eliminates the bias of having divided the training set in such a way that it favors a specific classification model.

## 6. Results

This section presents the IoT device built in this paper, a suitable outlier detection algorithm, and the classification model to be allocated in the device to make decisions locally. To define each algorithm, we carried out several tests with the dataset and used classification metrics, such as: accuracy, sensitivity, specificity, and recall, using the confusion matrix method. The classification algorithms were first trained with the original data set to determine the improvement in outlier detection techniques. Then, the same process was performed using the refined databases. For the deep learning method, a part of the training subset was considered, along with a validation set used to determine learning parameters and avoid over-training or unnecessary use of layers and neurons. This also allows for simplification of the model. Finally, this section shows the monitoring interface performed in the Cloud.

### 6.1. Sensor Calibration

**Figure 4:**
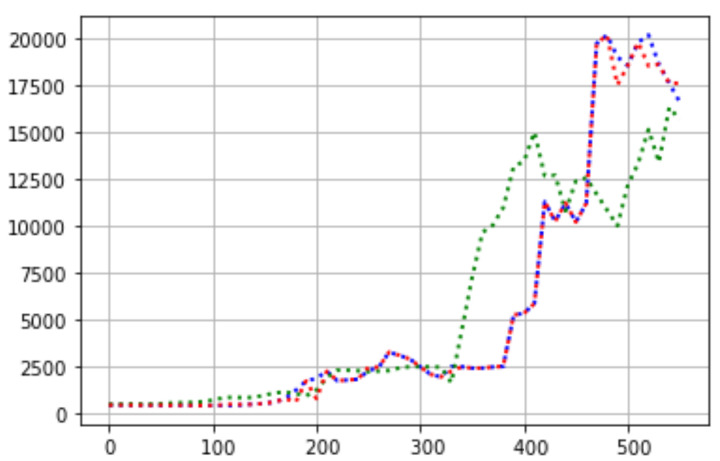
Regression models analysis from CO in ppm. Dotted dashed line in blue color: Result of the measurements of the reference grade instrument. Dotted dashed line in green color: Signal provided by the CO sensor. Dotted dashed line in red color: Result of the prediction algorithm based on the decision tree regressor. Finally, the abscissa axis represents the number of samples of the CO signal.

Different sensors are currently available in the market, which are characterized by the following: small size, low power requirements, and cost. However, their accuracy might be influenced by environmental factors and particle properties. Therefore, low-cost sensors need a reference-grade or research-grade instrument. Consequently, regression and ML methods are trained to obtain calibration formulas and test them using statistical metrics [37]. Therefore, the reference-grade instrument used was a Testo 315-3 (https://www.testo.com/en-US/testo-315-3/p/0632-3153 accessed on 17 August 2022), which simultaneously measures CO and CO${}_{2}$ in the environment of heating installations and outlets. Then, in a controlled environment, the Testo 315-3 and the IoT device collect data to determine the difference in the measurements. Next, regression models are trained with the aim that the IoT replicates the reference-grade instrument samples. Graphically, we determined that linear regression models are not suitable solutions by using statistical metrics such as the mean absolute error (MAE), root mean squared error (RMSE), and R-squared ($R^{2}$) [32]. Therefore, SVM, decision tree, and random forest were tested [31]. Table 2 shows the results obtained from CO and how the decision tree regressor pushes the samples of the IoT device so that they can fit into the samples obtained from the reference-grade instrument (e.g., $R^{2}=0.99$). Finally, Figure 4 shows the predictions of the decision tree regressor method. It can be observed that the predictions made are satisfactory; the measurements carried out by the sensor are transformed into something that is comparable with the measurements carried out by a reference instrument.

With these results, the values of each sensor were compared with different free access applications (Plume Labs (https://plumelabs.com/en/ accessed on 17 August 2022) and iqair (https://www.iqair.com/ accessed on 17 August 2022)) and robust detection systems that provide these variables. A 5% variability was achieved between the reference values and those obtained by the sensors, demonstrating the reliability of the sensors.

### 6.2. IoT Device

**Figure 5:**
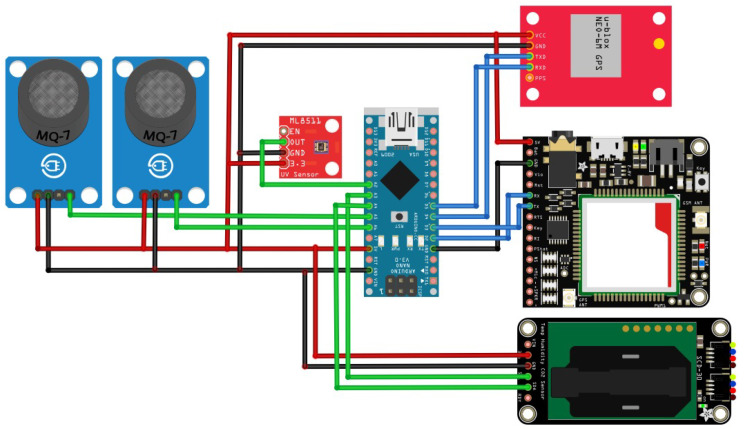
System circuit diagram. Voltage supply (–), Ground (–), Sensors communication (–), and Expansion board and GPS/GPRS sensor (–).

VLM6075 and SCD30 sensors use I${}_{2}$C communication. For this reason, they can only be connected to the SCL and SDA pins of the microcontroller. In addition, the GPS/GPRS sensor uses serial communication pins to send AT messages that allow its configuration. Finally, it is essential to mention that an expansion board has been implemented to possess an external memory to locally store data, in case of connection losses to the server. Figure 5 shows the electronic diagram, connecting sensors, microcontroller, and expansion board.

**Figure 6:**
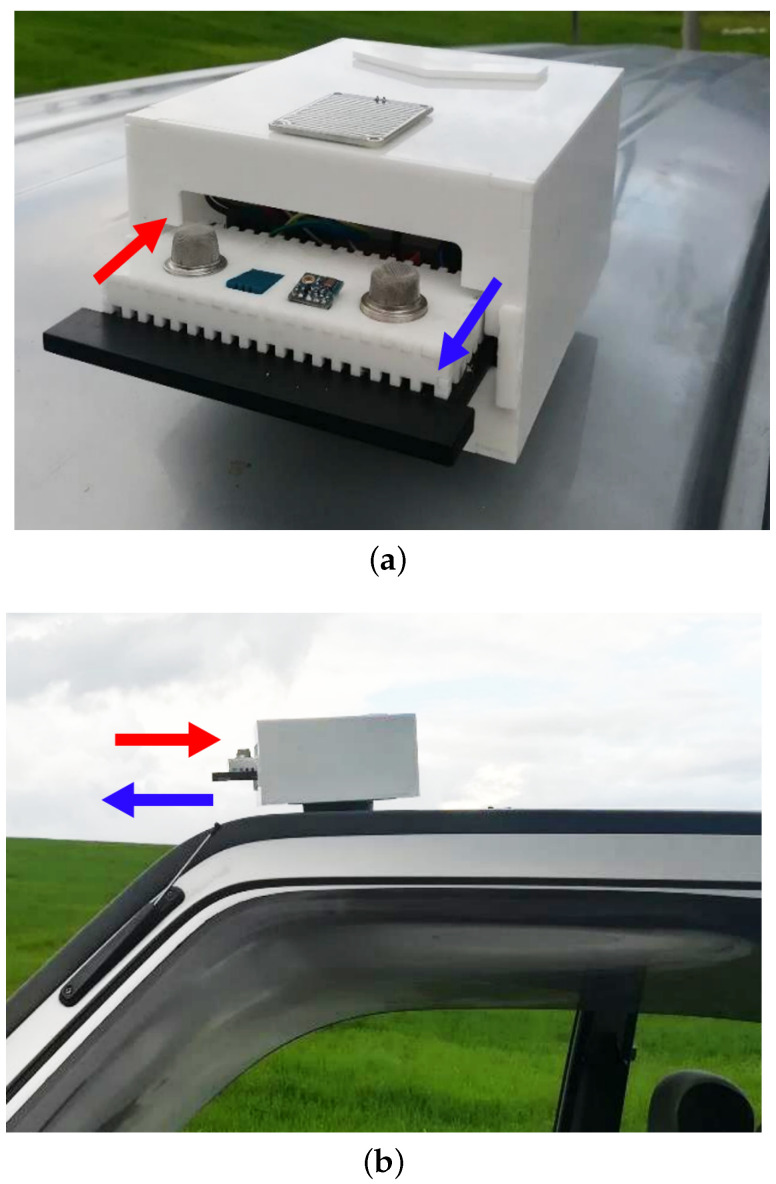
Prototype of the IoT device that was built for monitoring environmental conditions. (**a**) Waterproof case. (**b**) IoT device operating on the roof of the vehicle. Red arrow: The rain sensor detects drops on the waterproof case and activates the mechanism to save the sensors. Blue arrow: The car engine powers on and activates the IoT device, which gets out from the waterproof case.

The IoT device is designed to operate outside the vehicle used in this research (see Figure 6). In addition, at the top of the rain protector case, it has a rain sensor that, when activated, causes the system to retract inside the waterproof case and prevents the sensors from directly contacting water. Additionally, the sensors go into power-saving mode to avoid erroneous readings. The IoT device is configured to take samples every minute because it is the average time measured in real tests of how long a vehicle takes to travel roughly 250 m in a city with traffic. Each IoT system has an identifier that is indifferent to the car in which it is placed. Figure 6 shows both the waterproof case that was designed for the system and the installation of the system in the vehicle.

### 6.3. Data Analysis

The algorithms used in the outlier detection techniques and supervised classification models have been established. These models are developed to determine their classification performance. Datasets were randomly divided ten times into training sets and test sets to train ML models, where 10% of the samples were part of the test set. The metrics used were as follows: accuracy, sensitivity, specificity, and recall using the confusion matrix method. It is important to remember that pruned datasets are temporarily stored to train ML models, then just the outlier detection method will prune incoming data. As shown in Table 3, where we present the accuracy of each model, the SVM algorithm performs well below expectations despite using different kernel functions. On the other hand, the Naive Bayes algorithm has an average performance of 75%, which indicates that it has errors in differentiating between labels. However, decision trees, k-NN, and neural networks have suitable performances for describing the studied phenomenon. Nevertheless, k-NN is a slow algorithm, because it needs to carry out comparisons over the whole training set to find the nearest neighbors. For this reason, this algorithm cannot be employed in the IoT device because its memory consumption and response time will be very high. On the other hand, decision trees and neural networks show high classification performance. Consequently, these two models were exported to the IoT device to validate their performance in real conditions. Finally, the One-Class SVM algorithm is a suitable outlier detection technique, since it improves the classification performance and reduces the dataset by 10%.

### 6.4. Processing the Inference On-Device

With the IoT device installed, the inference of ML models is exported to test their performance and classification capacity in real environments. It is worth mentioning that the neural network has an input layer of six neurons due to the variables to be measured. The neural network has hidden layers of 20 neurons, 12 neurons, and 6 neurons, respectively, and an output layer of 3 neurons corresponding to each label. This model was the one with the smallest architecture (i.e., number of layers and neurons) able to achieve a performance higher than 90%. In addition, the computational requirements for each algorithm are established. RAMco is the memory used to compile the code, and RAMex is the memory already used when using all the variables, taking into consideration that these values are the result of compiling both the outlier detection and the inference of the ML model. This information is shown in Table 4. As a result, the decision-tree algorithm has a lower computational cost and response time, but a lower classification performance concerning the neural network, demonstrating a better pattern recognition model to make a decision.

### 6.5. Monitoring Interface

**Figure 7:**
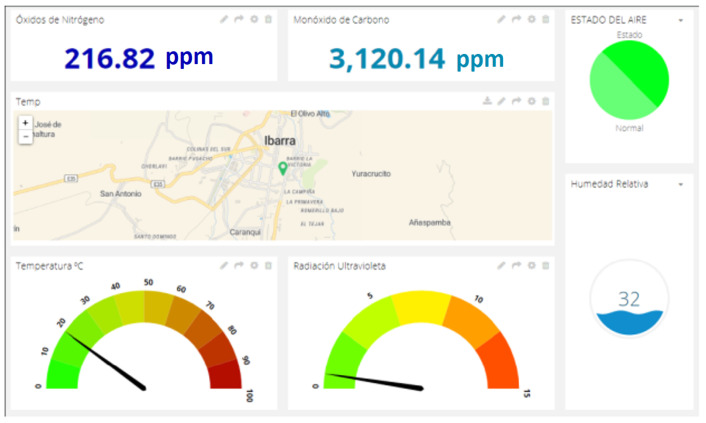
Cloud-hosted monitoring interface.

**Figure 8:**
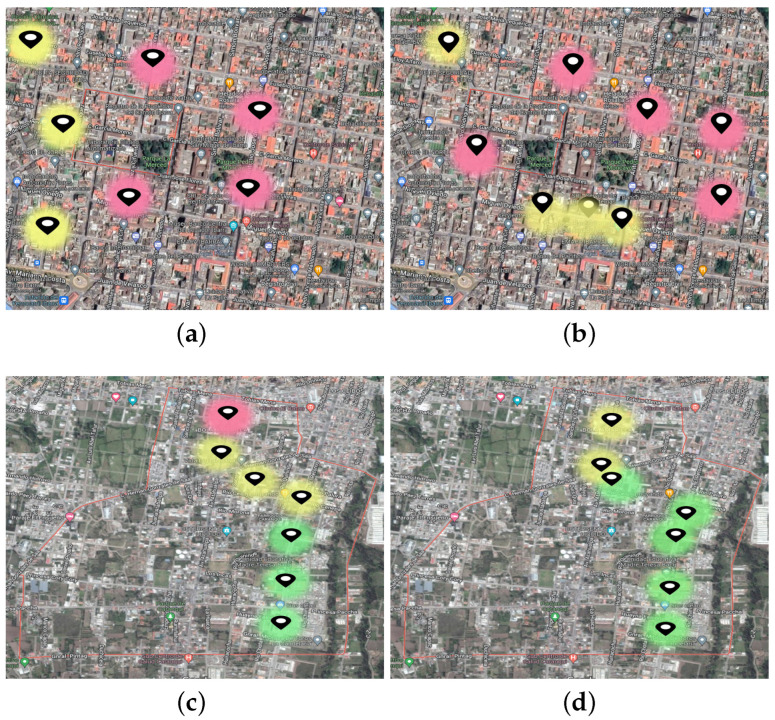
Visualization of data on environmental conditions in the city of Ibarra, in accordance with the defined pollution traffic light (see Section 2.1). (**a**) Downtown Ibarra: Morning. (**b**) Downtown Ibarra: Afternoon. (**c**) Uptown Ibarra: Afternoon. (**d**) Uptown Ibarra: Night.

IoT devices are working and, when the air pollution changes (e.g., from label C to B), they send data to the MQTT edge server, which stores the information in InfluxDB. From a cloud viewer, queries are made to the database for report generation. Figure 7 shows air-pollution information obtained from a point in the city, seen on a dashboard. In addition, the dashboard demonstrates that the environmental conditions are adequate. Furthermore, Figure 8 shows a real example of taking samples when vehicles move around the city centre. Figure 8a,b show the case in which several nodes transmit information at the same time. Moreover, Figure 8c,d show the case where a single vehicle moves and the environmental conditions are changing in different city zones.

### 6.6. Power Consumption Analysis

Guaranteeing the required power consumption and bandwidth needed is of paramount importance. Therefore, the IoT device is divided into three execution types:

- Processing time (the system is working).
- Sending processing time (the system activates the GPS/GPRS sensor and sends data to the Edge).
- Resting time (the system is in sleep mode, saving battery).

Then, the power consumption is defined according to the execution types. The processing time consumes 90 mA per second (microcontroller and sensors), the sending processing time consumes 50 mA (GPS/GPRS sensor is sending data to the Edge), and the resting time consumes 190 μA due to all the system is in sleep mode. Traditionally, the IoT device sends data each minute to the Edge, where the processing time and the sending processing time work simultaneously for 10 s, and the rest of the time, the device sleeps. Therefore, the power consumption per hour is 7800 mA. In addition, the inference working into the IoT device sends data only when the air-pollution variables change according to the previously defined labels (see Section 5.1). Therefore, the IoT device sends data each 4 min even when the processing time is working. As a result, the IoT device can collect data while maintaining the GPS/GPRS sensor inactive, and the power consumption decreases to 6000 mA.

Following the same execution times, the bandwidth is needed to send data each minute, with the 32 bytes as payload (i.e., identifier, NO${}_{x}$, CO, temperature, humidity, CO${}_{2}$, longitude, and latitude). Consequently, each hour, a single IoT device sends 1920 bytes. Furthermore, when taking the decision locally, the system sends data approximately each 4 min, which reduces to 480 bytes per hour. Moreover, this optimization of the data sent improves the bandwidth when the number of IoT devices increases.

## 7. Conclusions

This research aimed to develop a smart, portable air-quality monitoring system that can make decisions locally about air-pollution conditions in Ibarra. In addition, we present an adequate IoT architecture with a lightweight messaging protocol, the MQTT and a time series database allocated in the Edge. As a result, the IoT device can classify three different pollution levels and represent them in a heat map. Therefore, areas with a higher concentration of gases in Ibarra were established and, with in-depth subsequent inspections, it was found that some of these areas were created by the accumulation of buses and deficient synchronization of traffic lights in the city, which affect vehicular traffic. In short, the conclusions are presented as follows:

- Selecting the most representative air-pollution gases and sensors, with the capacity to read data, is a key stage in developing an air-quality IoT application.
- Outlier detection is essential in the IoT system, because it allows the classification algorithm to make accurate decisions. In addition, it was possible to show that the system has errors when the vehicles that have the IoT node installed travel behind the buses. This is because the exhaust pipe sometimes emits polluting gases directly into the electronic system, producing outliers. After that, the values are quickly reduced. However, when several buses are stopped and the system detects that the concentration of the buses fits the established model, it changes its decision to a high pollution level, since the concentration of gases is maintained.
- The proposed architecture of an IoT-oriented communication protocol and nonrelational time series database allows for optimization of the communication channel and suitable sizing of storage. Therefore, using a visualization tool in the Cloud with permanent queries to edge computing represents a comprehensive solution for IoT applications. Nevertheless, it leaves new challenges regarding the reliability of information from the Cloud to IoT devices, and how this can affect decision making on large data volumes.

In future works, we look forward to implementing cameras and robust ML analysis to both improve the obtained results and test the maximum number of allowed sensors.

## Figures and Tables

**Table 1:** Gases and sensors that were used in relevant related works.

Author	uC	Prot. Comm.	Sensed Parameters								Sensors
			CO	NO${}_{x}$	Temp.	Hum.	CO${}_{2}$	SO${}_{2}$	O${}_{3}$	Others	
[2]	Wasp-mote	ZigBee	x	x	x	x	x	x	x		Gasense Pro Libelium
[9]	Atmega 328p	ZigBee	x	x	x	x	x		x	CI${}_{2}$	GP2Y1, DSM501, MQ-7
[22]	ESP8266	WiFi	x					x			CH${}_{4}$MQ-7, MQ-4, MQ-135
[27]	LoRa-Arduino	LoRa WAN	x		x	x	x				MQ-7, MQ-4, MQ-135, DTH22
[17]	ESP32	WiFi	x	x	x	x	x	x			MH-Z14, MICS-4514, DHT22
[12]	Murata	LoRa WAN	x	x	x	x	x				Alphasense CO-B4, NO2-B43F
[16]	Arduino Mega	LTE			x	x	x	x			No mention
[28]	–	–			x	x				PM${}_{1}$ PM${}_{2.5}$ PM${}_{10}$	AirBeam2, DustTrak
[29]	STM32	–	x	x	x	x	x	x	x		T6615-5KF, SHT21, HPM 115S0-XXX
[3]	Arduino Mega 2560	LTE	x	x	x	x	x			PM${}_{1}$ PM${}_{2.5}$	MiCS-2714, MiCS-4514, MQ131, SPS30, BME280, DGS-O3/NO${}_{x}$/CO

**Table 2:** CO (ppm) regression analysis.

Algorithms	MAE	RMSE	$R^{2}$
IoT device samples	2256	3803	0.66
SVM	2239.12	3852.77	0.66
Random forest	849.51	1552.50	0.94
Decision Tree	114.05	368.50	0.99

**Table 3:** Classification accuracy results summary after using outlier detection techniques.

Data Base	Size	SVM	Tree	k-NN	Bayes	Neural Network
Original	560 Mb	0.33	0.85	0.84	0.70	0.85
Local	510 Mb	0.29	0.92	0.90	0.80	0.92
Isolation	525 Mb	0.38	0.87	0.86	0.79	0.96
One-Class SVM	515 Mb	0.25	0.98	0.96	0.68	0.96

**Table 4:** Computational requirements of ML models in the IoT device.

Algorithms	Flash	RAMco	RAMex	T. Response	Acc
Decision Tree	11,500	430	2805	0.65 μs	0.90
Neural Network	14,280	520	2910	0.91 μs	0.92

## Data Availability

Not applicable.
